# Effects of Ascent to High Altitude on Human Antimycobacterial Immunity

**DOI:** 10.1371/journal.pone.0074220

**Published:** 2013-09-13

**Authors:** Sarah Eisen, Louise Pealing, Robert W. Aldridge, Mark J. Siedner, Alejandro Necochea, Inna Leybell, Teresa Valencia, Beatriz Herrera, Siouxsie Wiles, Jon S. Friedland, Robert H. Gilman, Carlton A. Evans

**Affiliations:** 1 IFHAD: Innovation For Health And Development, Laboratory of Research and Development 218, Universidad Peruana Cayetano Heredia, San Martin de Porres, Lima, Peru; 2 Research Department of Infection and Population Health and the School of Medicine, University College London, London, United Kingdom; 3 Innovacion Por la Salud Y el Desarollo (IPSYD), Asociación Benefica Prisma, Lima, Peru; 4 Yale School of Medicine, New Haven, Connecticut, United States of America; 5 Infectious Diseases & Immunity, Imperial College London and Wellcome Trust Imperial College Centre for Global Health Research, London, United Kingdom; 6 Department of International Health, Johns Hopkins Bloomberg School of Public Health, Baltimore, Maryland, United States of America; McGill University, Canada

## Abstract

**Background:**

Tuberculosis infection, disease and mortality are all less common at high than low altitude and ascent to high altitude was historically recommended for treatment. The immunological and mycobacterial mechanisms underlying the association between altitude and tuberculosis are unclear. We studied the effects of altitude on mycobacteria and antimycobacterial immunity.

**Methods:**

Antimycobacterial immunity was assayed in 15 healthy adults residing at low altitude before and after they ascended to 3400 meters; and in 47 long-term high-altitude residents. Antimycobacterial immunity was assessed as the extent to which participants’ whole blood supported or restricted growth of genetically modified luminescent Bacille Calmette-Guérin (BCG) mycobacteria during 96 hours incubation. We developed a simplified whole blood assay that could be used by a technician in a low-technology setting. We used this to compare mycobacterial growth in participants’ whole blood versus positive-control culture broth and versus negative-control plasma.

**Results:**

Measurements of mycobacterial luminescence predicted the number of mycobacterial colonies cultured six weeks later. At low altitude, mycobacteria grew in blood at similar rates to positive-control culture broth whereas ascent to high altitude was associated with restriction (p≤0.002) of mycobacterial growth to be 4-times less than in culture broth. At low altitude, mycobacteria grew in blood 25-times more than negative-control plasma whereas ascent to high altitude was associated with restriction (p≤0.01) of mycobacterial growth to be only 6-times more than in plasma. There was no evidence of differences in antimycobacterial immunity at high altitude between people who had recently ascended to high altitude versus long-term high-altitude residents.

**Conclusions:**

An assay of luminescent mycobacterial growth in whole blood was adapted and found to be feasible in low-resource settings. This demonstrated that ascent to or residence at high altitude was associated with decreased mycobacterial growth in whole blood relative to controls, consistent with altitude-related augmentation of antimycobacterial cellular immunity.

## Introduction

One third of the world’s population is estimated to be latently infected with tuberculosis (TB) and progression to TB disease causes 1.4 million deaths each year [1]. However, in the great majority of immunocompetent people latently infected with TB, antimycobacterial immunity prevents progression to symptomatic disease [1]. Furthermore, in the pre-antibiotic era many episodes of symptomatic TB disease were controlled by the host immune system and apparently resolved spontaneously [2]. Even with effective antibiotic treatment, the likelihood of treatment achieving a lasting cure is also influenced by host immunity [3,4]. Thus, TB infection represents a balance between progression of the mycobacterial infection and containment by antimycobacterial immunity. Antibiotic-resistant TB has increasing prevalence but limited diagnosis and treatment facilities and the emergence of TB strains resistant to almost all known antibiotics [5] is renewing interest in approaches to strengthen patients’ antimycobacterial immunity [6].

Altitude therapy and TB care in mountain sanatoria have been used historically for TB care [7] and epidemiological studies suggest that high altitude is associated with reduced incidence of TB infection, disease and mortality [8-17]. However, the mechanisms underlying this apparent altitude-mediated effect are poorly understood, and are likely to include both mycobacterial transmission and host antimycobacterial immunity [18].

Antimycobacterial immunity has been studied *in vitro* by quantifying the restriction of mycobacterial growth by immune cells [19,20]. This has been facilitated by the development of genetically modified luminescent mycobacteria that allow rapid and accurate mycobacterial quantification, overcoming their slow growth on solid media and inaccuracies caused by mycobacteria clumping [21,22]. A whole-blood model of the extent to which blood cells support or restrict the growth of luminescent mycobacteria has been used effectively for investigating aspects of human antimycobacterial cellular immunity [23]. These include correlation with cytokine production and tuberculin skin test status [24], evaluation of vaccine candidates [14,25] and of nutritional augmentation of antimycobacterial immunity [26].

To enable studies in a high altitude setting, we simplified previously published whole-blood assays of antimycobacterial immunity so that a technician in a low-technology setting with only portable equipment and without a biosafety cabinet, centrifuge or freezer could concurrently perform multiple assays. Since the antimycobacterial immunity assays were repeated at different times and locations, variations in temperature, oxygen tension, mycobacterial stocks, atmospheric pressure and other factors inevitably caused inter-assay differences in mycobacterial growth. To reduce the effect of these inter-assay variations in mycobacterial growth, antimycobacterial immunity was assessed as the extent to which participants’ whole blood supported or restricted mycobacterial growth relative to concurrent growth in positive-control culture broth. Humoral (acellular) immunity is relatively inactive against mycobacteria and plasma is generally tuberculostatic (neither supporting mycobacterial growth nor killing mycobacteria). Thus antimycobacterial immunity was also inferred from the extent to which whole blood supported mycobacterial growth relative to negative-control plasma.

We used this assay to assess the effects of high altitude on peoples’ antimycobacterial immunity.

## Methods

### Ethics statement

This study was approved by the Ethics Committees of the Asociación Benéfica PRISMA (Lima), and the Universidad Peruana Cayetano Heredia (Lima). All participants were fully informed in their preferred language (Spanish or English) about this study and gave signed written consent. Mechanisms were in place to provide a free chest radiograph, sputum testing and medical consultation to any participants with suspected TB and to refer any participants with symptoms suggestive of TB to the local National TB Control Program, which provides free TB diagnosis and treatment. All data collected and questionnaires completed were confidential and identified by means of a coding system rather than by the subject’s name.

### Subjects

**Figure 1:**
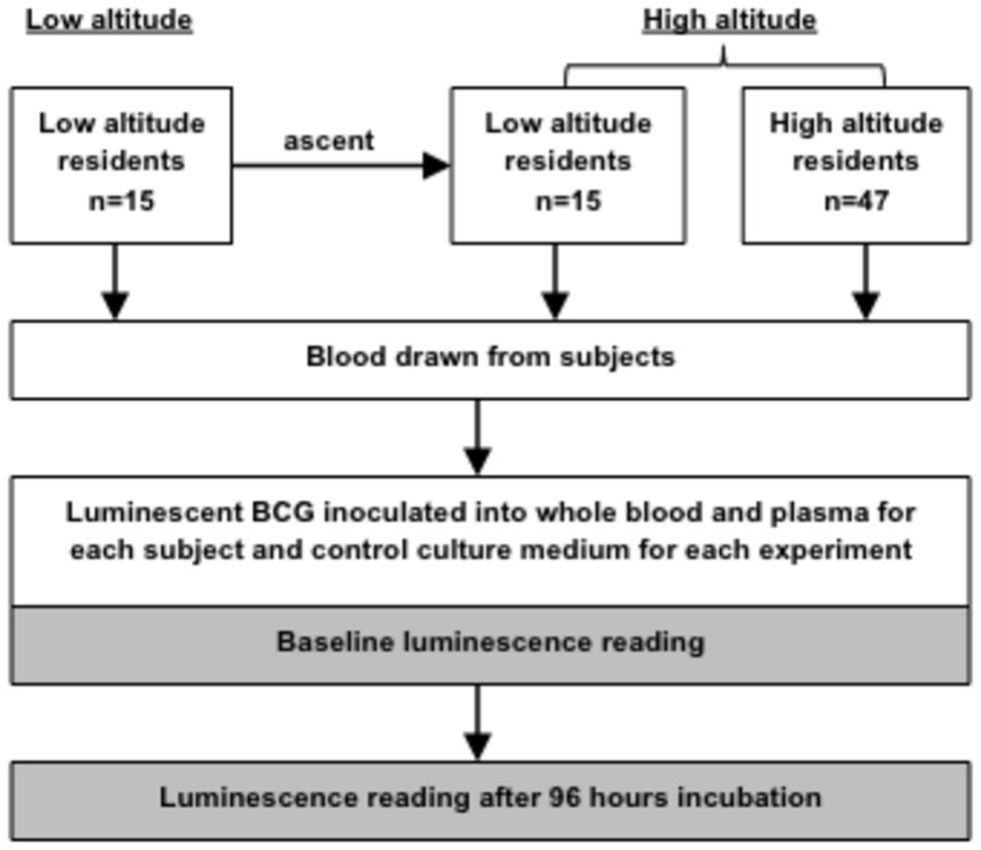
Study design. Fifteen low altitude residents had blood drawn at low altitude, which was used to obtain whole blood and plasma samples that, together with culture broth samples, were then inoculated with luminescent mycobacteria. In half of these samples, baseline luminescence readings were measured straight away. The other half of these samples were incubated at 37°C for 96 hours and luminescence readings were repeated. The low altitude residents ascended to high altitude and together with a group of 47 high altitude residents, blood samples were taken and the experiment repeated.

The effect of altitude on antimycobacterial immunity was studied by comparing samples collected under three conditions (Figure 1). Firstly, low-altitude residents living in Lima, Peru, less than 100 meters above sea level, provided an initial blood sample at low-altitude. Secondly, they then provided another sample after ascent to high altitude in Cusco, Peru, 3400 meters above sea level. Thirdly, samples were also obtained from permanent residents at the same high altitude location. Participants were identified by inviting university staff, affiliates and students to participate. Inclusion criteria were healthy adults who gave informed written consent for this research that had internationally accredited ethical committee approval. Exclusion criteria were travel to high altitude during the previous month or having a tuberculin skin test within the previous six months, in case these affected antimycobacterial immunity. A questionnaire was used to characterize demographic data, previous TB exposure and vaccination with bacille Calmette-Guérin (BCG). The nutritional status of the individuals was assessed by measuring mid-upper arm circumference, height, weight and calculating body mass index.

### Mycobacteria stocks

Luminescent *Mycobacterium bovis* BCG (Montreal strain) were used that were a gift from collaborators who had transformed them to constitutively express the *lux* gene as described [21]. BCG was used rather than virulent *Mycobacterium tuberculosis* for biosafety reasons. These mycobacteria were maintained by continuous passage in stock cultures in exponential growth in Middlebrook 7H9 culture broth supplemented with 10% growth supplement (oleic acid, albumin, dextrose and catalase, OADC, Becton Dickinson, Oxford, UK). All culture media were also supplemented with hygromycin (50 µg/mL; Roche, Lewes, UK) to select the genetically modified luminescent strain [22].

### Mycobacterial quantification

Mycobacterial stock cultures were quantified each time that they were required for experimental work and approximately twice weekly when growth became macroscopically visible, indicating that dilution was required to maintain exponential growth. Quantification was done by taking an aliquot to which substrate (1% n-decyl aldehyde in 100% ethanol, Sigma, MO, USA) was added for immediate measurement of luminescence in an automated portable luminometer (Turner 2020, Turner designs, CA, USA). The base-ten logarithms (log_10_) of the luminometer readings (in arbitrary relative light units) had an approximately Gaussian distribution and are referred to throughout this manuscript as “luminescence”. In baseline studies, serial logarithmic dilutions of the stock broth cultures and concurrent cultures in blood and plasma were quantified by luminescence and then immediately plated on Middlebrook 7H11 culture agar containing OADC supplement. These were incubated for six weeks and then the number of colonies was counted. This confirmed that luminescence was linearly related to the number of mycobacterial colonies cultured six weeks later, as reported previously [24]. Thus the measurement of luminescence determined the concentration of these genetically modified mycobacteria in less than one minute instead of the weeks that are generally required for quantifying most non-luminescent mycobacteria.

### Mycobacterial inoculum

For each assay, an aliquot of the mycobacterial stocks that were maintained in exponential growth was diluted with phosphate-buffered saline at pH 6.8 (Sigma-Aldrich, St Louis, MO, USA) until its luminescence indicated a mycobacterial concentration equivalent to 740 mycobacterial colonies per 100 µL (see below). This concentration was selected so that after dilution in whole blood for the antimycobacterial immunity assay, the concentration of mycobacterial colony forming units would be approximately one per 30 monocytes. The actual multiplicity of infection depended upon the subject’s blood monocyte concentration: approximately one colony forming unit per 10 monocytes if the subject’s monocyte concentration was at the lower limit of the normal range; or one colony forming unit per 50 monocytes if the subject’s monocyte concentration was at the upper limit of the normal range. After the pilot experiments that determined and optimized these concentrations, human blood monocytes were neither quantified nor purified for the research described below. Thus the actual cell counts at low and high altitude were not determined.

### Blood samples

At least 1.5 ml of venous blood was collected into heparinized tubes (LH-Metal-Analytik, Sarstedt, Numbrecht, Germany) and kept at 37°C in a portable incubator (App-Chem Ltd, Wrexham, UK) until analysis. One ml of blood was centrifuged to obtain plasma (the liquid part of blood with cells removed). Volumes of 450 µL of each subject’s blood and of their plasma were supplemented with equal volumes of diluent (RPMI 1640 with 1% HEPES N-2-hydroxyethylpiperazine-N’-2-ethanesulfonic acid; Life Technologies Ltd, Paisley, UK) to stabilize pH and provide nutrients for blood cells (where present) during incubation for all the procedures described below.

### Antimycobacterial immunity assays

The mycobacterial inoculum (100 µL) was mixed with 900 µL aliquots of blood, positive-control culture broth and negative-control plasma. One positive-control culture broth specimen was analyzed together with each batch of approximately 10 participants’ blood/plasma samples that were analyzed together. Mycobacterial concentrations were quantified in all aliquots immediately after inoculation as a baseline and in identical concurrently prepared aliquots after they had been incubated for 96 hours at 37°C in room air. Analysis of mycobacterial concentrations was done by addition of nine volumes of sterile water to reduce opacity (and lyse blood cells where present), followed by the addition of substrate for measurement of luminescence as described above.

#### (a): Mycobacterial growth

The raw data characterizing the growth of mycobacteria in each of blood, positive-control culture broth and negative-control plasma were calculated as the luminescence readings after 96-hours incubation minus the baseline pre-incubation luminescence readings.

#### (b): Antimycobacterial immunity

Antimycobacterial immunity was assessed as the extent to which participants’ whole blood supported or restricted mycobacterial growth relative to concurrent growth in positive-control culture broth (growth during 96 hours incubation in culture broth minus concurrent growth in blood). Antimycobacterial immunity was also inferred from the extent to which whole blood supported mycobacterial growth relative to negative-control plasma (growth during 96 hours incubation in blood minus concurrent growth in plasma).

### Quadruplet assays

To determine the reproducibility of assay results, all the above baseline and 96-hour measurements in blood, positive-control culture broth and negative-control plasma were performed using quadruplet identical tubes. Results were analyzed using only the data from the first of the quadruplet tubes and also using the average of the quadruplet tubes.

### Data analysis and statistical methods

All statistical tests were two-tailed and used STATA version 11 software (STATA Corporation, College Station, Texas). Fisher’s exact test was used for categorical variables. Data were not normally distributed so non-parametric tests were used: the Wilcoxon rank sum test for unpaired data and the Wilcoxon signed-rank test for paired low altitude versus high altitude comparisons in each individual.

## Results

### Subjects

Fifteen low-altitude residents were studied at low altitude and again an average of 11.1 (standard deviation 6.4) hours after ascent to high altitude. Additionally, 47 permanently high-altitude residents were studied only at this high altitude. The demographic and anthropometric characteristics of these participants are shown in Table 1. The low-altitude residents were older than the high-altitude residents (26 versus 22 years respectively, p<0.001). There was no evidence of differences in nutritional indicators (body mass index, upper arm circumference), sex or rates of previous BCG vaccination (all p≥0.4). No participants had previously been diagnosed with TB disease.

**Table 1:** Characteristics of study participants.

Characteristic	Low altitude residents	High altitude residents	p-value
	N=15	N=47	
Male %	47	55	1.0
[95% confidence intervals]	[18,75]	[41,70]	
(n/N)	(7/15)	(26/47)	
Age median years	26	22	<0.001
(interquartile range)	(24, 31)	(20, 23)	
BCG scar seen %	71	46	0.4
[95% confidence intervals]	[44,98]	[30,61]	
(n/N)	(10/14)	(20/44)	
Body mass index median kg/m2	21	21	0.6
(IQR)	(19, 23)	(20, 23)	
Mid upper arm circumference (median cm)	27	26	0.7
(interquartile range)	(23, 29)	(25, 28)	

Abbreviations: N, group size; n, number.

#### (a): Mycobacterial growth

The mycobacterial growth results are shown in Table 2a. At low and high altitude, mycobacteria grew abundantly in positive-control culture broth, minimally in negative-control plasma and there was intermediate mycobacterial growth in whole blood. Ascent from low to high altitude was associated with increases in mycobacterial growth in culture broth (p<0.001) and plasma (p=0.004) but not in whole blood (p=0.3). High altitude increased mycobacterial growth similarly in culture broth and in plasma, so there was no evidence of any difference in mycobacterial growth in culture broth relative to plasma comparing low versus high altitude results (p≥0.2).

**Table 2:** Altitude effects on luminescence indicating mycobacterial growth* in blood, positive-control culture broth and negative-control plasma.

	*Mycobacterial* growth				p-values		
	Low altitude residents at low altitude	Low altitude residents at high altitude	High altitude residents at high altitude		Low altitude residents: change on ascent to high altitude	At high altitude: low altitude residents versus high altitude residents	Low altitude residents at low altitude versus high altitude residents at high altitude
	(n=15)	(n=15)	(n=47)				
(a) MYCOBACTERIAL GROWTH							
Culture broth	1.1	1.9	1.5		<0.001	<0.001	<0.001
	[0.70, 1.1]	[1.9, 2.3]	[1.5, 1.7]				
Whole blood	0.86	1.2	0.95		0.3	0.7	0.5
	[0.53, 1.2]	[0.30, 1.4]	[0.48, 1.4]				
Plasma	-0.55	0.40	0.094		0.004	0.9	<0.001
	[-0.64, -0.37]	[-0.59, 0.70]	[-0.15, 0.44]				
Culture broth relative to plasma	1.5	1.4	1.5		0.2	0.3	0.8
	[1.3, 1.7]	[1.2, 2.8]	[1.2, 1.7]				
(b) ANTIMYCOBACTERIAL IMMUNITY							
Whole blood relative to culture broth	0.17	0.62	0.65		0.002	0.4	0.001
	[-0.10, 0.43]	[0.43, 2.0]	[0.26, 1.0]				
Whole blood relative to plasma	1.4	0.76	0.79		0.01	1.0	0.002
	[1.1, 1.4]	[0.50, 1.0]	[0.46, 1.2]				

* Median [interquartile range] increase in luminescence measured in log_10_ relative light units in each medium. Unpaired data comparisons were made with Wilcoxon rank sum test and paired data comparisons were made with the Wilcoxon signed-rank test.

### (b): Antimycobacterial immunity

**Figure 2:**
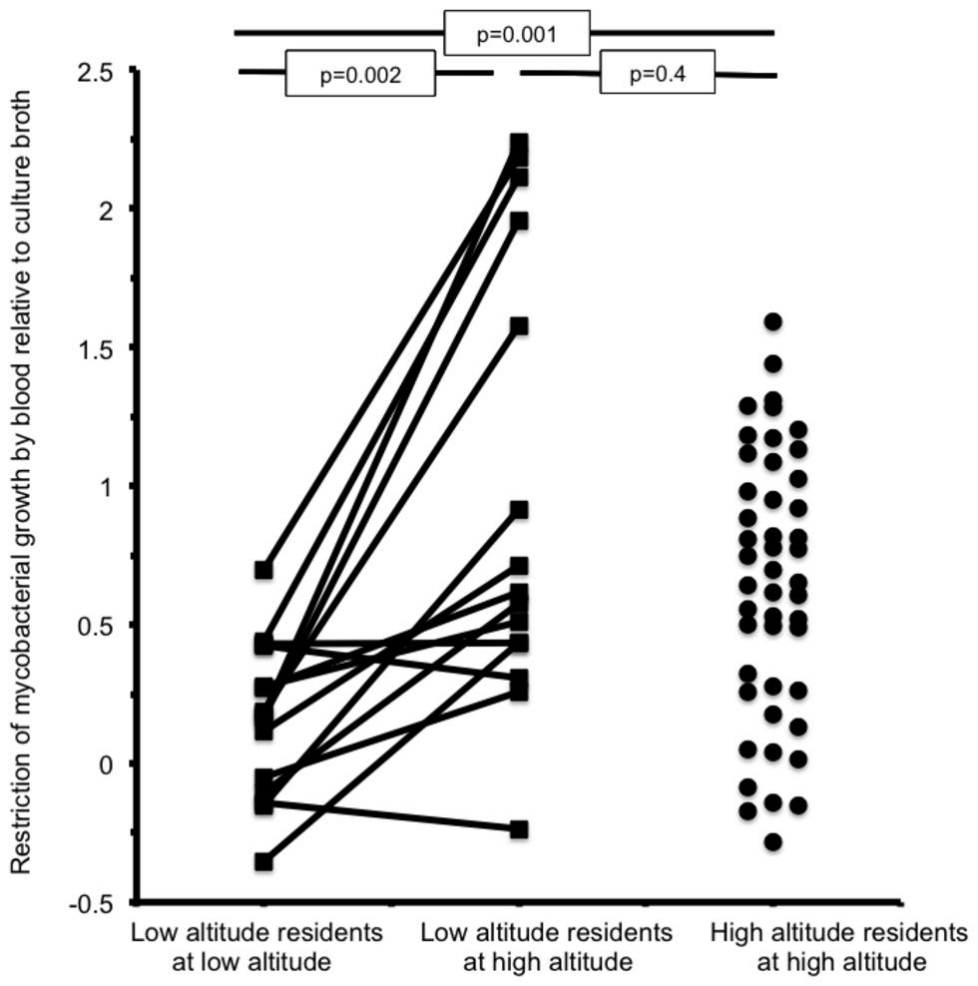
Restriction of mycobacterial growth by blood relative to culture broth.

The restriction of mycobacterial growth by whole blood relative to positive-control culture broth is shown in Table 2b and in Figure 2. At low altitude, mycobacteria grew similarly abundantly in blood as in the positive-control culture broth (p=0.4). Specifically, mycobacterial growth in blood was only 0.17 logs (i.e. 1.5-times) less than growth in positive-control culture broth. In contrast, ascent to high altitude was associated with restriction (p=0.002) of mycobacterial growth in the same participants’ blood to be 0.62 logs (i.e. 4-times) less abundant than the positive-control culture broth. Blood from long-term high-altitude residents also restricted mycobacterial growth to be 0.65 logs (i.e. 4-times) less abundant than the positive-control culture broth (p=0.001 versus low altitude residents at low altitude).

 Antimycobacterial immunity was assessed in 15 low altitude residents as they ascended to high altitude (square symbols joined by lines) and in 47 high altitude residents at high altitude (circular symbols). The vertical axis shows luminescence recorded as the log_10_ of measured relative light units (arbitrary units) produced by genetically modified luminescent mycobacteria. The increase in luminescence over 96 hours incubation is shown positive-control culture broth minus whole blood. For low altitude residents at low altitude, mycobacteria grew similarly in culture broth and blood. Ascent to high altitude was associated with restriction of mycobacterial growth in whole blood relative to culture broth, similar to the values for high altitude residents at high altitude.

The extent to which whole blood supported mycobacterial growth relative to negative-control plasma is also shown in Table 2b. At low altitude, mycobacteria grew 1.4 logs (i.e. 25-times) more abundantly in participants’ whole blood than in their negative-control plasma. In contrast, ascent to high altitude was associated with restriction (p=0.01) of mycobacterial growth in the same participants’ blood to be only 0.76 logs (i.e. 6-times) more abundant than their negative-control plasma.

There was no evidence of differences in antimycobacterial immunity at high altitude between people who were usually low-altitude residents versus long-term high-altitude residents (Table 2b). This was true whether considering growth in whole blood relative to positive-control culture broth (p=0.4) or considering growth in whole blood relative to negative-control plasma (p=1.0).

### Age

Because of the difference in age between low and high-altitude residents, we tested for an association between participant age and the results of the antimycobacterial immunity assay. Whether age was analyzed as a continuous variable or a dichotomous variable (split by median age), and whether analyzing all 77 assay results or only the 62 results for assays performed at high altitude, there was no evidence of an association between age and the antimycobacterial immunity assay results (all p>0.4).

### Quadruplet tubes in each assay

In contrast with the marked changes in mycobacterial growth under different experimental conditions, there was minimal variation in luminescence assessments of mycobacterial concentrations between the identical quadruplicate tubes that made up each assay (data not shown). Consequently, repeating the above analyses using the average of the results from the quadruplet identical tubes instead of the first tube did not alter the study findings nor their statistical significance (see supporting information Table S1).

Some of the results reported in this manuscript have been previously reported in the form of an abstract [27].

## Discussion

In this study we simplified an assay of human antimycobacterial immunity to study altitude effects. This demonstrated the feasibility of this assay for assessing antimycobacterial immunity in low-resource settings and revealed opposing effects of high altitude on the pathogen versus the host. Specifically, ascent to high altitude both facilitated the growth of the mycobacterial pathogen and appeared to augment healthy participants’ immune resistance to this pathogen. This antimycobacterial immunity was assessed as the restriction of mycobacterial growth in whole blood relative to positive-control culture broth and was also inferred from the mycobacterial growth in whole blood relative to negative-control acellular plasma.

High altitude settings have historically been associated with apparent protection from TB [7] and people diagnosed with TB still sometimes travel to high altitude because they believe that this will cure their disease [28]. There is epidemiological evidence from several countries that increasing altitude is associated with decreased rates of TB latent infection [8,9] and morbidity [12,14,15,29]. Even after adjusting these epidemiological analyses for potentially confounding variables, high altitude was associated with decreased rates of TB disease [10,11,13,14] and death [16,17]. Several of these studies were located in Peru, where the current research also took place [8,9,15]. In the pre-antibiotic era these and other factors led to patients with TB disease being treated with ascent to and supportive care provided at high altitude, so-called “altitude therapy” for TB [7]. Rigorous evaluation of this approach appears to be lacking and it has largely fallen into disuse since the advent of effective antimycobacterial antibiotics.

The association between increasing altitude and reduced TB might imply that high altitude directly impairs mycobacterial growth. We found the opposite: that ascent to high altitude was in fact associated with increased mycobacterial growth in both culture broth and human plasma. Human antimycobacterial immunity is largely cellular and consequently plasma would not be expected to mount effective dynamic antimycobacterial immune responses and plasma has long been known to be tuberculostatic, neither supporting TB growth nor killing TB [30]. The effect of altitude on extracellular mycobacterial growth in plasma does not appear to have been defined previously, and this part of our findings is consistent with studies of aeration and oxygenation that, depending upon experimental conditions, may stimulate or impair extracellular mycobacterial growth [31-33]. Thus, high altitude stimulated extracellular mycobacterial growth in human plasma under the experimental conditions that we used.

In the current research, altitude-mediated augmentation of extracellular mycobacterial growth in plasma was counteracted by findings consistent with altitude-mediated augmentation of cellular antimycobacterial immunity. This finding was apparent for mycobacterial growth in whole blood compared with both the positive-control culture-broth in each assay and also the negative-control plasma from each individual. We have not been able to identify previous human studies of altitude and antimycobacterial immunity. However, our novel finding is supported by experiments in: animals in which altitude augmented antimycobacterial immunity [34]; in cells in which hypoxia stimulated the human vitamin D-dependent antimycobacterial pathway [32]; and in humans whose non-specific cellular immunity was augmented by altitude [35,36]. These findings demonstrate increased immune resistance to mycobacteria at altitude, which offers an explanation for the apparent success of altitude therapy for TB. Other non-immunological factors that may also contribute to reduced TB transmission (and thus disease) at high altitude include germicidal effects of altitude-associated ultraviolet light [37], reduced humidity [38], and hypoxia [33].

Our objective to study the effect of altitude on human antimycobacterial immunity was challenged by factors that we addressed by developing a simplified assay of antimycobacterial immunity. Firstly, the complexity of published antimycobacterial immunity assays limited their feasibility in high-altitude settings [19-22,24-26], so we simplified an assay to require only portable equipment: hand-held incubator, pipettes; and a briefcase sized luminometer. Secondly, mycobacterial quantification is often inaccurate, technically demanding and usually requires many weeks [19,20], so we used genetically-modified luminescent mycobacteria [21,22,24-26] and simplified their processing to quantify them reliably and instantly, without centrifugation. Thirdly, large-volume blood samples discourage participation; so we scaled the assay to require less than 1.5 ml of blood. Fourthly, we found in preliminary studies that day-to-day and place-to-place variations in mycobacterial growth confounded assessments of how well blood restricted mycobacterial growth, an issue that was likely to worsen in the extreme environments involved in our research. Consequently, we interpreted assays as relative mycobacterial growth in blood compared with positive-control culture-broth included in each assay and also relative to concurrent negative-control plasma from each individual. We used 1.5 ml of blood per participant but found high replicability between quadruplicate assays, so recommend that in future only single (not quadruplicate) assays be performed, which would require only 0.3 ml (6 drops) of blood. This simplified, low blood volume assay that uses only portable equipment should facilitate assessment of human antimycobacterial immunity in the resource-poor settings where most TB occurs [6,25,26,39].

Limitations to this study include the lack of adjustment for differences between the low and high altitude residents, such as their age. However, we found no evidence of an association between participant age and the results of the antimycobacterial immunity assay. Furthermore, these methodological limitations would not affect our experiment in which antimycobacterial immunity was assessed in the same individuals prior to and after ascent to high altitude. The logistical challenges of transferring research participants and the team and equipment for assessing antimycobacterial immunity from low to high altitude restricted the number of participants in this part of the research. This small sample size limits the certainty of our research findings that should be replicated in a larger study. A potentially affordable strategy for addressing this limitation in future research may be to re-assay antimycobacterial immunity again in each participant after returning to low altitude. It is also noteworthy that there is no gold standard, validated *in vitro* correlate of antimycobacterial immunity because no test has been shown to predict peoples’ resistance or susceptibility to TB infection or disease [25]. Thus our finding that ascent to high altitude increased cellular restriction of mycobacterial growth is consistent with but cannot provide absolute confirmation for the long-reported altitude-associated resistance to TB infection, disease and mortality.

The field use of this assay in a low-resource high-altitude setting limited our capacity for: (1) mechanistic studies of cell counts, cell types and immunological mediators and processes, so future studies should store frozen aliquots of the assays to characterize mediators of high altitude-augmentation of antimycobacterial immunity and to determine whether altitude directly augmented cellular anti-mycobacterial immunity or whether indirect mechanisms such as altitude-mediated changes in the mycobacterial and/or cellular nutrients or cell counts in blood may have contributed [36]; (2) confirming that luminescence predicted mycobacterial growth at high altitude as it did in validation experiments at low altitude by ourselves and by others [24] and that the altitude-associated effects on mycobacterial luminescence indicated mycobacterial killing not reduced metabolism or senescence; (3) using virulent *M. tuberculosis* instead of low-virulence BCG mycobacteria that is a relatively safe but imperfect surrogate for *Mycobacterium tuberculosis*; and (4) studying the time course of altitude-mediated augmentation of antimycobacterial immunity. It was noteworthy that we found similar antimycobacterial immunity less than one day after ascent to altitude as after long-term residence there. Larger studies may differentiate the potentially contributory mechanisms of altitude *per se* [36] versus associated hypoxia [18], possible increased exposure to sunlight increasing vitamin D levels [39] and nutritional changes [26].

These findings do not imply that patients with difficult-to-treat TB should migrate to high altitude, where we found that not only the disease-resisting human immunity but also the disease-causing pathogens were potentiated. However, our findings provide some evidence for an altitude-mediated augmentation of human antimycobacterial immunity that may explain the anecdotal observation that TB patients often improve and may even be cured when they move to high altitude and the well-proven rarity of TB at high altitude. A so-called “antibiotic apocalypse” of emerging resistance of *Mycobacterium tuberculosis* to almost all known antibiotics [40,41] has been reported. Thus, our findings justify rigorous evaluation of how altitude affects antimycobacterial immunity as this may be exploited to develop new treatments [6,32]. Clinical research is also needed to determine whether, as was common practice in the pre-antibiotic era, the increasing numbers of patients with TB resistant to all antibiotics would benefit from their care being moved to high-altitude [40,41].

## Supporting Information

Table S1 Altitude effects on luminescence indicating mycobacterial growth* in blood, positive-control culture broth and negative-control plasma. The average of data from quadruplet identical tubes for each assay are shown.
(DOCX)
