# Supplementary material for: Effects of Ascent to High Altitude on Human Antimycobacterial Immunity
Source: PLoS One. 2013 Sep 13;8(9):e74220. doi: 10.1371/journal.pone.0074220 (PMC3772817; doi:10.1371/journal.pone.0074220)
Supplement: Table S1 — Altitude effects on luminescence indicating mycobacterial growth* in blood, positive-control culture broth and negative-control plasma. The average of data from quadruplet identical tubes for each assay are shown. (DOCX) [file pone.0074220.s001.docx]

**Supporting information**

| Table S1. Altitude effects on luminescence indicating mycobacterial growth* in blood, positive-control culture broth and negative-control plasma. The average of data from quadruplet identical tubes for each assay are shown. | | | | | | | |
| --- | --- | --- | --- | --- | --- | --- | --- |
|  | *Mycobacterial* growth | | |  | p-values | | |
|  | Low altitude residents at low altitude | Low altitude residents at high altitude | High altitude residents at high altitude |  | Low altitude residents: change on ascent to high altitude | At high altitude: low altitude residents versus high altitude residents | Low altitude residents at low altitude versus high altitude residents at high altitude |
|  | (n=15) | (n=15) | (n=47) |  |  |  |  |
| (a) MYCOBACTERIAL GROWTH | |  |  |  |  |  |  |
| Culture broth | 1.3 | 1.9 | 1.5 |  | <0.001 | <0.001 | <0.001 |
|  | [0.63, 1.4] | [1.9, 2.3] | [1.5, 1.7] |  |  |  |  |
| Whole blood | 0.89 | 1.2 | 1.0 |  | 0.1 | 0.9 | 0.4 |
|  | [0.54, 1.0] | [0.51, 1.4] | [0.58, 1.5] |  |  |  |  |
| Plasma | -0.58 | 0.32 | 0.064 |  | 0.004 | 0.8 | <0.001 |
|  | [-0.70,-0.17] | [-0.61, 0.66] | [-0.15, 0.38] |  |  |  |  |
| Culture broth relative to plasma | 1.4 | 1.6 | 1.5 |  | 0.3 | 0.4 | 0.4 |
|  | [1.3, 2,0] | [1.2, 2.7] | [1.2, 1.7] |  |  |  |  |
|  |  |  |  |  |  |  |  |
| (b) ANTIMYCOBACTERIAL IMMUNITY | |  |  |  |  |  |  |
| Whole blood relative to culture broth | 0.36 | 0.64 | 0.67 |  | 0.001 | 0.1 | 0.02 |
|  | [-0.070,0.50] | [0.41, 1.6] | [0.16, 0.90] |  |  |  |  |
| Whole blood relative to plasma | 1.4 | 0.86 | 0.78 |  | 0.009 | 0.8 | 0.003 |
|  | [1.1, 1.5] | [0.43, 0.99] | [0.56, 1.3] |  |  |  |  |

*Median [interquartile range] increase in luminescence measured in log_10_ relative light units in each medium. Unpaired data comparisons were made with Wilcoxon rank sum test and paired data comparisons with the Wilcoxon signed-rank test.
